# Measurement of Scattering and Absorption Cross Sections of Dyed Microspheres

**DOI:** 10.6028/jres.118.002

**Published:** 2013-01-14

**Authors:** Adolfas K Gaigalas, Steven Choquette, Yu-Zhong Zhang

**Affiliations:** 1 National Institute of Standards and Technology, Gaithersburg, MD 20899; 2 Life Technologies, 29851 Willow Creek Rd., Eugene, OR 97402

**Keywords:** absorption, dye, fluorescence quantum yield, integrating sphere detector, Lorenz-Mie scattering, microspheres, scattering

## Abstract

Measurements of absorbance and fluorescence emission were carried out on aqueous suspensions of polystyrene (PS) microspheres with a diameter of 2.5 µm using a spectrophotometer with an integrating sphere detector. The apparatus and the principles of measurements were described in our earlier publications. Microspheres with and without green BODIPY^@^ dye were measured. Placing the suspension inside an integrating sphere (IS) detector of the spectrophotometer yielded (after a correction for fluorescence emission) the absorbance (called *A* in the text) due to absorption by BODIPY^@^ dye inside the microsphere. An estimate of the absorbance due to scattering alone was obtained by subtracting the corrected BODIPY^@^ dye absorbance (*A*) from the measured absorbance of a suspension placed outside the IS detector (called *A_1_* in the text). The absorption of the BODIPY^@^ dye inside the microsphere was analyzed using an imaginary index of refraction parameterized with three Gaussian-Lorentz functions. The Kramer-Kronig relation was used to estimate the contribution of the BODIPY^@^ dye to the real part of the microsphere index of refraction. The complex index of refraction, obtained from the analysis of *A*, was used to analyze the absorbance due to scattering ((*A_1_*- *A*) in the text). In practice, the analysis of the scattering absorbance, *A_1_-A*, and the absorbance, *A*, was carried out in an iterative manner. It was assumed that *A* depended primarily on the imaginary part of the microsphere index of refraction with the other parameters playing a secondary role. Therefore *A* was first analyzed using values of the other parameters obtained from a fit to the absorbance due to scattering, *A_1_-A*, with the imaginary part neglected. The imaginary part obtained from the analysis of *A* was then used to reanalyze *A_1_-A*, and obtain better estimates of the other parameters. After a few iterations, consistent estimates were obtained of the scattering and absorption cross sections in the wavelength region 300 nm to 800 nm.

## 1. Introduction

Several previous papers from our group discussed the measurement of fluorescent quantum yield (QY), and light scattering and absorption cross sections using a spectrophotometer with an integrating sphere detector [1,2]. In references 1 and 2 the measurement of scattering cross section for polystyrene (PS) microspheres was discussed. The PS microspheres had characteristic strong absorption for wavelengths below 300 nm and vanishingly small absorption for wavelengths above 300 nm. The measurements were analyzed assuming that the measured absorbance below 300 nm was due to styrene absorption, and the absorbance above 300 nm was due to scattering of photons from the incident light beam. The two results were combined to describe the scattering and absorption cross sections from 240 nm to 800 nm. The correspondence between the predicted and measured values was very good. The present work deals with measurements of absorption and scattering from monodisperse PS microspheres which contain green BODIPY^@^ fluorophores embedded in the polystyrene matrix (dyed PS microspheres). The dyed microspheres have maximum absorption around 571 nm where there is also strong scattering. Our present work suggests that it is possible to measure the BODIPY^@^ absorption by placing the suspension inside an integrating sphere (IS) detector, and then combine the measured absorption with the independent measurement of the total absorbance, with the suspension placed outside the IS detector, to obtain the absorption and scattering cross sections for wavelengths between 300 nm and 800 nm. The measurement of dye absorption with the suspension inside the integrating sphere detector required a correction for dye fluorescence which yielded an estimate of the microsphere fluorescence quantum yield.

## 2. Experimental Method

### 2.1 Microsphere Fabrication

**Fig. 1:**
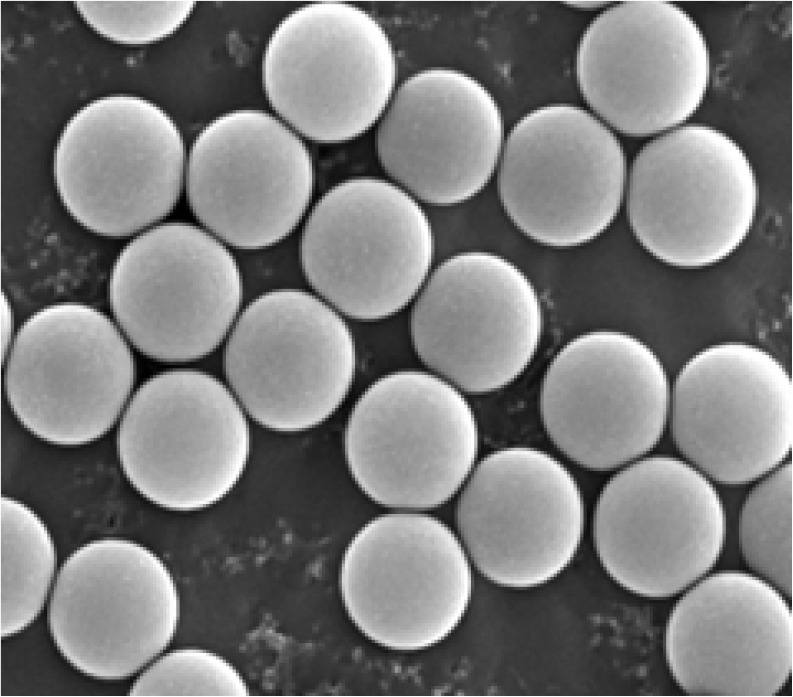
Transmission electron microscope image of the polystyrene microspheres used in this study. Similar pictures were obtained after the incorporation of green BODIPY^@^ dye into the polystyrene matrix. The diameter and the coefficient of variation (CV) were 2.5 µm and 5.2 % respectively for both the dyed and un-dyed microspheres.

The fabrication of monodisperse microspheres has been described in detail in reference [3]. Some of the unique features used in the production of the dyed microspheres used in this work are outlined below. Monodisperse polystyrene microspheres used in this research were prepared by seed emulsion polymerization of styrene in a surfactant-free reaction system. The particles were spherical with mean diameter and coefficient of variation equal to 2.5 µm and 5.2 %, respectively. The size was determined from transmission electron microscopy (TEM) images one of which is shown in Fig 1.

The green fluorescent BODIPY^®^ dye (Molecular Probes, US Patent # 4,774.339)^1^ was incorporated into the 2.5 µm polystyrene microspheres using a proprietary solvent system to generate the green fluorescent microspheres. With this unique method, the molecules of green BODIPY^®^ dye were incorporated uniformly throughout the polymer matrix that provided a highly hydrophobic environment suitable to the organic dye [4]. The dye molecules were oriented randomly in the polymer matrix [5,6]. The diameter of the fluorescent microspheres remained the same as that of the plain polystyrene beads as indicated by the TEM measurement of the dyed microspheres. The QY of the green fluorescent BODIPY^®^ dye in ethanol is given in the literature as 0.95 and it will be used in the analysis of measurements [7]. The suspensions used in the measurements described below were prepared by pipetting several µL of stock suspension of dyed microspheres obtained from Life Technologies, Inc., into 10 mL of distilled water.

### 2.2 Measurement Details

**Fig. 2:**
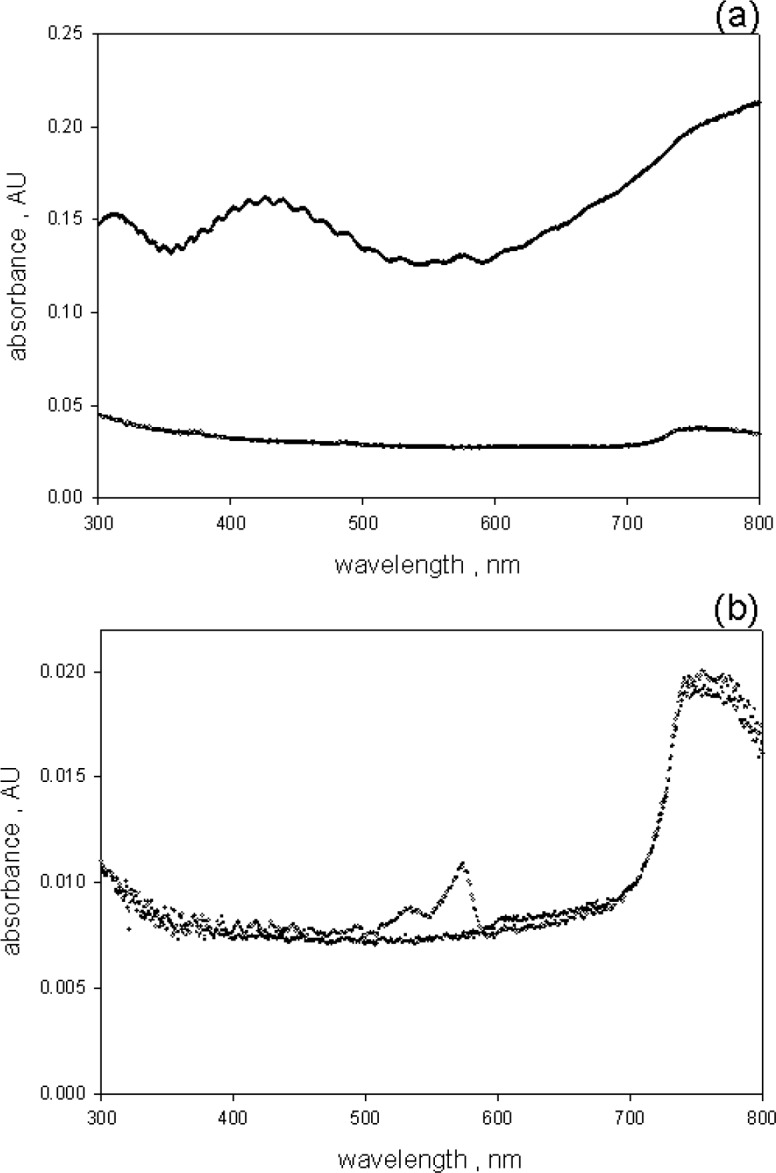
(a) The upper trace shows the absorbance measured for a suspension of microspheres placed in spectrometer holder 1 which is located outside the integrating sphere (IS) detector. The lower trace shows the measurement for water. The water measurement was subtracted from the suspension measurement prior to analysis. (b) The two traces show the absorbance of suspension and water samples placed inside the IS detector. The suspension trace exhibits an absorption peak at 571 nm.

Absorbance measurements were performed with the Lambda 850 spectrometer equipped with a 150 mm integrating sphere (IS) detector. The configuration of the sample holders and the light beams is shown in Fig. 1 in reference [1]. The Lambda 850 spectrometer was scanned from 800 nm to 300 nm in increments of 1 nm with an integration time of 0.52 s, and a slit width of 2 nm. Four measurements were taken with water and the suspension placed sequentially in holder 1(outside IS) and holder 3 inside IS). The upper and lower traces in Fig. 2a show the measured absorbance of samples of microsphere suspension and water placed in holder 1 and 2 respectively. The upper and lower traces in Fig. 2b show the measured absorbance of the same samples of microsphere suspension and water placed in holder 3 respectively (*A_3_*). The absorbance measured in holder 3 for water and the microsphere suspensions were very similar except around 571 nm where the BODIPY^@^ dye absorbs. (The measured absorbance in holder 3 was very small and care had to be taken to avoid artifacts, as large as 0.002 AU, due to cuvette orientation and cuvette cleanliness.) The absorbance due to scattering was obtained by subtracting the measured absorbance in holder 3 (after correction for fluorescence) from the measured absorbance in holder 1 (*A_1_*) as described in a previous paper [1]. In both cases the absorbance measured in water was subtracted from the measured absorbance of the microsphere suspension. The preliminary data subtraction was performed with Mathcad. The Lorenz-Mie calculations were performed using MatLab with Maetzler [8] code for Lorenz-Mie scattering.

Fluorescence measurements were carried out with a SLM 8000 fluorimeter. The slits of the illumination and detector monochromators were set to 8 nm, the excitation was 555 nm, the integration time was set to 1 s. Spectral response corrections were not performed.

## 3. Measurement of Absorbance Due to Dye Absorption

### 3.1 Measurement of Molecular Absorption in Scattering Suspensions

**Fig. 3:**
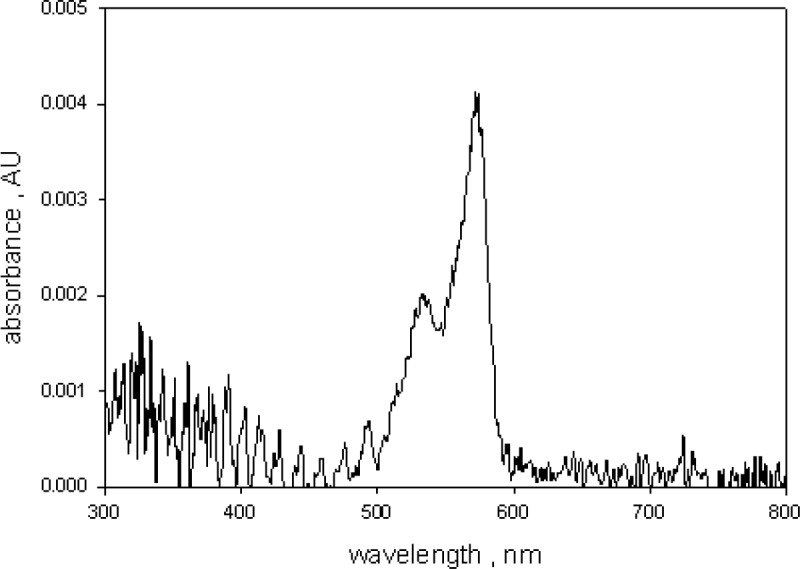
The absorbance of the microsphere suspension after subtraction of the absorbance of suspension of undyed microspheres. The absorbance above 600 nm is approximately zero. The ripples below 500 nm were due to small difference in the diameters of the dyed and the undyed microspheres. A suspension of undyed microspheres resulted in a better background subtraction than a sample of water. The uncertainty in the peak absorbance value is about 5 %.

The comparison of absorbance measurements in holder 1 and holder 3 showed that the measurements in holder 3 were not very sensitive to scattering losses. There was a small background absorbance (of the order of 0.003 absorbance units) which was most likely due to the escape of backward scattered photons through the entrance aperture of the IS detector. The scattering background observed in holder 3 was relatively constant over the wavelength region between 600 nm and 800 nm and this region was used to estimate the scattering background. However there was a gentle rise in the scattering background in the region of BODIPY absorption which made it difficult to subtract the background using water as the reference for this spectral region. The trace in Fig. 3 shows the result of measurements of absorbance in holder 3 using a suspension of 2.5 µm blank(not dyed) microspheres as a reference and subtracting from measurements of dyed microspheres. The scattering background is minimal for wavelengths greater than 550 nm and shows a ripple structure for wavelengths less than 550 nm. The ripples are originated from small difference in the diameters of the dyed and blank 2.5 µm microspheres. The small differences in diameters lead to a small shift in the scattering pattern resulting in an incomplete subtraction of scattering signals from the two microsphere suspensions. The trace in Fig. 3 may still not represent the true absorbance because the microspheres fluoresce and the emitted fluorescence reduced the observed absorbance in Fig. 3.

### 3.2 Fluorescence Correction of Absorbance Measurements in Holder 3

**Fig. 4:**
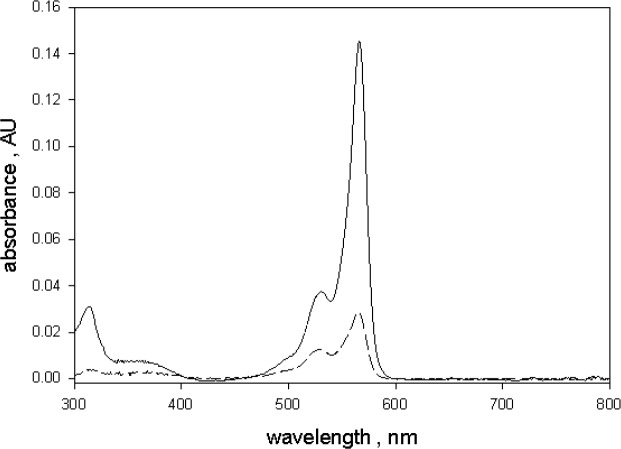
The two traces show the absorbance of a 10 µmol/L solution of BODIPY^@^ dye in ethanol. The solid and dashed traces were obtained for samples outside (called *A_1_* in the text) and inside (called *A_3_* in the text) the integrating sphere (IS) detector respectively. The large decrease in the absorbance for the sample inside the IS detector is due to the emitted fluorescence. The IS detector cannot distinguish between transmitted and fluorescence photons, and interprets the fluorescence photons as transmitted photons.

**Fig. 5:**
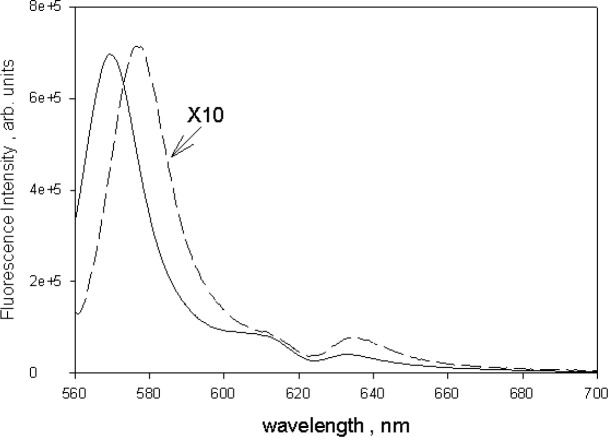
The solid trace shows the fluorescence emission spectrum of 10 µmol/L solution of BODIPY^@^ dye in ethanol excited with 555 nm light. The dashed trace shows the fluorescence emission from a suspension of microspheres dyed with BODIPY^@^ dye and excited with 555 nm light. The wavelength of maximum microsphere emission is shifted to the red by about 9 nm relative to the maximum emission of BODIPY^@^ dye in ethanol. In both case, the strong fluorescence emission reduced the absorbance measured for samples inside the IS detector.

Figure 4 shows the effect of fluorescence on absorbance measurements of a sample of 10 µmol/L green BODIPY^®^ dye dissolved in ethanol and placed in holder 3. The solid and dashed traces in Fig. 4 show the absorbance measurements carried out in holder 1(outside IS) and holder 3 (inside IS) respectively. Measurements were also done for a sample of ethanol to subtract ethanol contribution. The absorbance measured in holder 1 is a good estimate (after subtracting the ethanol contribution) of the true BODIPY^@^ absorption. The reduction of the green BODIPY^®^ dye absorbance observed in holder 3 was attributed to the fluorescence emitted by the excited green BODIPY^®^ dye molecules. The spectrophotometer detector could not distinguish between transmitted and fluorescent photons and any fluorescence photon was interpreted by the instrument as a transmitted photon. A closer examination of the two traces in Fig. 4 also displayed a large difference in spectral shape. The spectral shape of the measurement in holder 3 was influenced by the spectral response of the detector. Most of the fluorescence photons were emitted around 570 nm while the wavelength of the transmitted photons varied in accordance with the spectrometer setting. Thus the measurements in holder 1 and holder 3 have a different effective detector spectral response. These considerations also apply to absorbance measurements of microspheres dyed with the green BODIPY^®^ dye. The dashed trace in Fig. 5 shows the emitted fluorescence spectrum from a suspension of 2.5 µm dyed microspheres placed in a fluorimeter and excited with 555 nm light. For comparison, the solid trace in Fig. 5 shows the observed fluorescence emission when an ethanol solution of green BODIPY^®^ dye was placed in a fluorimeter and excited with 555 nm light. A comparison of the two traces in Fig. 5 shows that the fluorescence emission from the microspheres is similar to the fluorescence emission from the dye solution except for a red shift of approximately 9 nm. Therefore it is reasonable to assume that the observed absorbance of 2.5 µm microsphere suspension, shown in Fig. 2, would be reduced by the emitted fluorescence and that the actual absorbance of the microsphere suspension is larger. In order to quantify the reduction of the measured absorbance due to fluorescence, it was assumed that the relative reduction in absorbance is proportionate to the quantum yield as indicated in Eq. (1).

(1) $$\frac{A-A_{3}}{A}=m\Phi$$

**Fig. 6:**
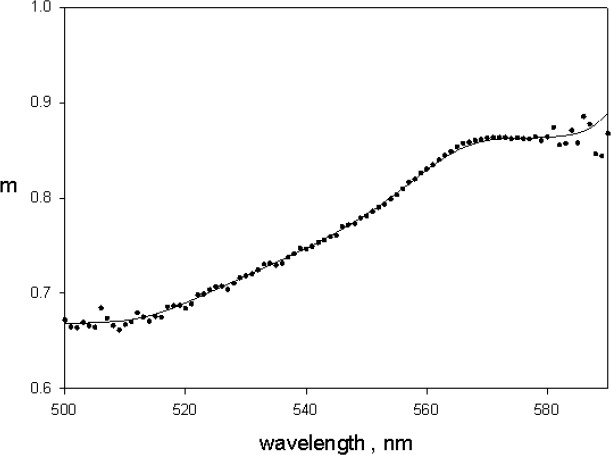
The solid circles show the correction factor *m* obtained using Eq. (2) in the text and the absorption measurements shown in Fig. 4 (*A_1_* outside the IS and *A_3_* inside the IS). The fluorescence quantum yield of BODIPY^@^ dye in ethanol was set to 0.95 in Eq. (2).

Here *A* is the true absorbance, *A_3_* is the absorbance measured in holder 3, Φ is the quantum yield, and *m* is proportionality constant. In the ideal case where every photon is detected with the same probability, the value of *m* should be 1 since in the ideal case the ratio on the left side of Eq. (1) is just the definition of the quantum yield. (*A* is a measure of absorption, *A_3_* is a measure of absorption without subsequent emission, hence *A-A_3_* is a measure of absorption with subsequent emission). In practice, the photon detection probability varies significantly across the wavelength region so that the value of *m* will be different from 1 as discussed in a previous work [9]. The absorbance measurements with the green BODIPY^®^ dye solution placed in holder 1 were used to estimate the true absorbance of green BODIPY^®^ dye, while the quantum yield of green BODIPY^®^ dye in ethanol has been determined to be 0.95 [7]. Explicitly, the proportionality constant *m* was assumed to be given by Eq. (2).

(2) $$\frac{A_{1}-A_{3}}{A_{1}}=m\cdot 0.95$$

where *A_1_* and *A_3_* are the absorbance measurements of BODIPY^@^ dye in ethanol obtained in holders 1 and 3 as shown in Fig. 4. Figure 6 shows the result of applying Eq. (2) to the data in Fig. 4. The value of *m* was well defined only in the region of large absorbance. Further from the absorbance maximum at 571 nm, the noise level becomes very large and values of *m* could not be evaluated with confidence. From the trace in Fig. 6 it is clear that the quantity *m* in Eq. (2) is not a constant and varies from 0.65 to 0.9 over the green BODIPY^®^ dye absorption band. The variation in the value of *m* was attributed to changes in detector spectral response. For measurements in holder 1 the detected wavelength is the same as that indicated by the instrument setting. In holder 3, the detected wavelength is a mix of indicated wavelength and fluorescence wavelength.

The observed fluorescence emission spectra of ethanol solution of BODIPY^®^ dye and the dyed microspheres were sufficiently similar so that the values of *m* obtained for the ethanol solution were directly applicable to the analysis of dyed microsphere measurements. However, in order to use Eq. (1) to correct the measured absorbance of dyed microspheres it was first necessary to estimate the quantum yield (QY) of dyed microsphere fluorescence. The QY of dyed microsphere fluorescence was determined at 555 nm. This choice of wavelength was motivated by the desire to minimize systematic errors in the vicinity of 555 nm. The quantum yield of microspheres was obtained relative to the quantum yield of BODIPY^®^ dye in ethanol by using the relation given in Eq. (3) [10].

(3) $$\frac{I}{I_{\mathrm{Et}}}=\frac{A}{A_{\mathrm{Et}}}\frac{\Phi }{\Phi_{\mathrm{Et}}}\frac{n^{2}}{n^{2}{}_{\mathrm{Et}}}$$

*I* and *I_Et_* are the integrated fluorescence intensities obtained by integrating the dashed and solid traces shown in Fig. 5 respectively. *A* and *A_Et_* are the absorption values of the microsphere suspension and green BODIPY^®^ dye ethanol solution respectively at the excitation wavelength of 555 nm. The indices of refraction *n* and *n_Et_* refer to water and ethanol respectively. The square of the ratio of the two indices was set to 1.046 in Eq. (3). Equation (3) indicates that the value of *A* is needed to find the microsphere quantum yield Φ, while Eq. (1) states that Φ is needed to determine the true microsphere absorption *A*. Solving Eq. (3) for Φ in terms of *A*, and substituting into Eq. (1) lead to an estimate of the true absorption of the dyed microsphere suspension at 555 nm and is given by Eq. (4). This result applies for the single wavelength which in this case is 555 nm.

(4) $$A=A_{3}+m\frac{I}{I_{\mathrm{Et}}}A_{\mathrm{Et}}\Phi_{\mathrm{Et}}$$

Inserting the various measured values into Eq. (4) resulted in *A* which is an increase in *A_3_* at 555 nm form 0.0024 to 0.0091. Inserting the corrected absorption of microspheres into Eq. (3) lead to a quantum yield of about 0.95 for the dyed microspheres. Repeated measurements for the dyed microspheres gave a quantum yield of 0.95±0.04. The quantum yield of green BODIPY^®^ dye molecules in dilute ethanol solution is 0.95, therefore there is an effective equality of fluorescence between dyed PS microspheres and dye in solution. The polystyrene matrix does not quench fluorescence from the green BODIPY^®^ dye.

Assuming that the quantum yield determined at 555 nm excitation remains constant for other wavelengths in the absorption band, Eq. (1) can be inverted to give Eq. (5) which corrects the entire absorbance spectrum.

(5) $$A=\frac{A_{3}}{1-m\cdot QY_{555}}$$

**Fig. 7:**
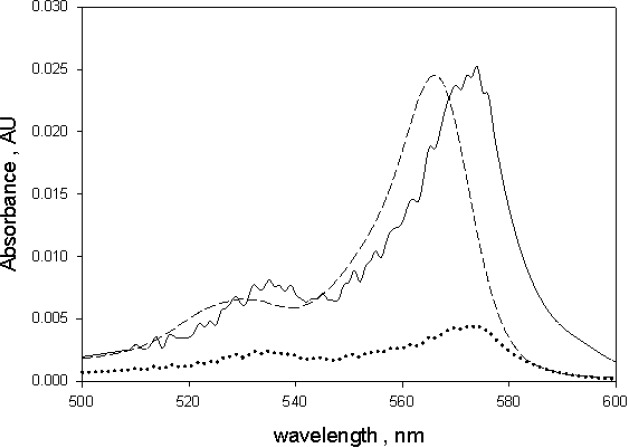
The dotted trace reproduces the trace from Fig. 3 and shows the absorbance of dyed microspheres as measured with the suspension inside the IS detector. The solid trace is the corrected absorbance using Eq. (1) in the text and the correction factor m given in Fig. 6. The quantum yield of the dyed microspheres was found using data in Fig. 5. The dashed trace is the normalized absorbance of BODIPY^@^ dye in ethanol. The spectral shapes of the absorbance in ethanol and polystyrene are very similar, however the spectrum of BODIPY^@^ dye in polystyrene is shifted to the red by about 9 nm.

Here *m* represents the vector of *m* values at wavelengths where *A_3_* was measured as shown in Fig. 6. The solid trace in Fig. 7 shows the result of applying Eq. (5) to the absorbance measurements shown in Fig. 2b. The dotted trace in Fig. 7 shows the original measured absorbance. The dashed trace in Fig. 7 shows the absorbance of green BODIPY^®^ dye in ethanol normalized to the maximum microsphere absorbance shown by the solid trace. The corrected spectral shape of the solid trace in Fig. 7 is similar to the spectral shape of green BODIPY^®^ dye in ethanol (dashed trace). The spectral distortion inherent in the measurements in holder 3 was corrected by the analysis given in Eq. (5). The wavelength of maximum microsphere absorption was shifted to the red by 9 nm relative to the wavelength of maximum absorption of green BODIPY^®^ dye in ethanol. Such shifts are observed when the same fluorophore is placed in different polar environments [11,12]. A similar relative red shift was also observed in the wavelengths of maximum fluorescence emission shown in Fig. 5.

### 3.3 Analysis of the Absorbance of Microspheres Dyed with Green BODIPY^@^

The solid trace in Fig. 7 was analyzed using the least square method given by Eq. (6).

(6) $$\begin{array}{l} Residuals=\sum_{\lambda }{\left(\frac{A}{c}-Mie(d,n)\right)}^{2}\\ \;c=N\cdot 0.01/2.303\\ \;N=microsphere\;concentration,\;ml^{-1}\\ \;d=diameter\\ \;n=index\;of\;refraction \end{array}$$

The output of the Lorenz-Mie code, *Mie*(*d*,*n*), [8] was set to give the absorption cross section which was then used directly in Eq. (6). The absorption cross section depends strongly on the imaginary part of the index of refraction, and depends to a lesser degree on the values of the other parameters. The real part of the index of refraction, and the diameter were set to the values characteristic of polystyrene microspheres obtained in previous scattering analysis. The imaginary component of the microsphere index of refraction was parameterized using three mixed Gaussian-Lorentz (GL) functions given in Eq. (7).

(7) $$\begin{array}{r} nimag(\lambda )=scale\cdot (GL(\lambda ,0.021,0.5715,0.8)\\ +0.24\cdot GL(\lambda ,0.032,0.534,1.0)\\ +0.06\cdot GL(\lambda ,0.120,0.334,1.0)) \end{array}$$

The wavelengths were in units of 10^−6^ m and the GL function was defined as shown in Eq. (8).

(8) $$GL(\lambda )=\frac{\exp(-2.773\cdot (1-mx)\cdot \frac{{\left(x-xp\right)}^{2}}{wd^{2}})}{1+4\cdot mx\cdot \frac{{\left(x-xp\right)}^{2}}{wd^{2}}}$$

**Fig. 8:**
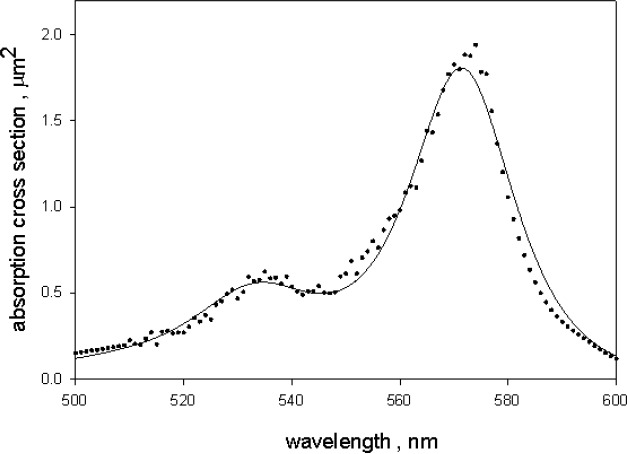
The solid circles show the absorption cross section of dyed microspheres. The values were obtained by dividing the corrected absorption (solid trace in Fig. 7) by the microsphere number concentration obtained from the fit to the scattering cross section. The solid trace is the calculated cross section using an imaginary index of refraction represented by three Gaussian-Lorentz peaks, and other parameters obtained from the fit to the scattering cross section.

The symbol *scale* is a parameter which was evaluated by requiring a best fit to the absorbance data, A/c, in Eq. (6). The parameters *xp* and *wd* define the peak location and the peak width respectively. The parameter *mx* is 0 for a pure Gaussian function and 1 for a pure Lorentz function. Intermediate values of *mx* give mixtures of the two functions. The parameters in the GL functions in Eq. (7) were chosen to best represent the measured absorbance. The solid circles in Fig. 8 give the values of *A*/*c* with the values of *A* taken from the solid curve in Fig. 7 and *c* determined by the best fit to scattering. The solid trace in Fig. 8 shows the absorption cross section which best fits the data with the value of the parameter *scale* equal to 0.0078.

The imaginary index of refraction, given by Eq. (7), originates from the presence of BODIPY dye in the microsphere, and we combined it linearly with the real part of the index of refraction of styrene to yield the total index of refraction of the microsphere. The linear combination was justified by the mixing relation given by Heller [13] to describe the total index of refraction of a two component mixture. The mixing relation is valid if the index of refraction of the bulk dye is close to the value of bulk styrene and if the concentration of the dye in the microsphere is very small. Since both conditions are satisfied, to a good approximation the total index of refraction of the microsphere is simply the sum of the part due to styrene and the part due to the dye. The BODIPY contribution to the real part of the microsphere index was estimated from the imaginary part given by Eq. (7) using the Kramers-Kronig relation shown in Eq. (9).

(9) $$\Delta nreal(\lambda )=-\frac{2\cdot \lambda^{2}}{\pi }\left(\int_{\delta }^{\lambda -\varepsilon }\frac{nimag(\lambda^{\prime })}{({\lambda^{\prime }}^{2}-\lambda^{2})\cdot \lambda^{\prime }}d\lambda^{\prime }+\int_{\lambda +\varepsilon }^{2}\frac{nimag(\lambda^{\prime })}{({\lambda^{\prime }}^{2}-\lambda^{2})\cdot \lambda^{\prime }}d\lambda^{\prime }\right)$$

**Fig. 9:**
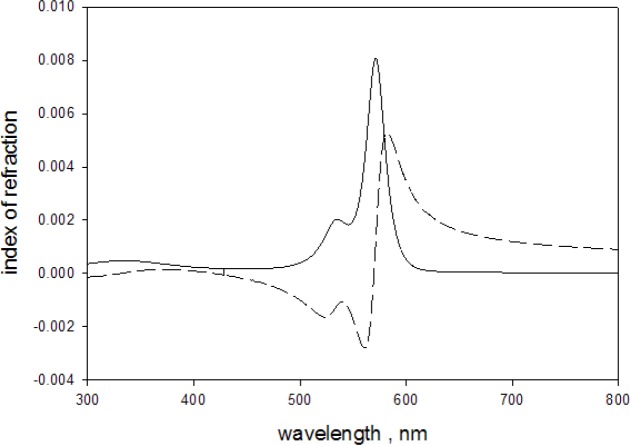
The solid trace shows the imaginary index of refraction obtained from the best fit to the absorbance data shown in Fig. 8. The dashed trace shows the corresponding real part of the index of refraction obtained from the Kramer-Kronig relation given in Eq. (9). The imaginary index is equal to the imaginary index of the microsphere since the styrene molecules do not absorb in this wavelength region. The real part is only 0.6 % of the total real index of refraction of the microsphere.

Equation (9) was obtained from Eq. 4.26 in Lucarini [14] by changing variables from angular frequency *ω* = 2·*π* · *c*_0_/*λ* to the wavelength, *λ*. Here *c_0_* and *λ* are the vacuum speed of light and the vacuum wavelength respectively. The two parameters in the integration limits in Eq. (9) were set to *δ* = 0.00001, and *δ* = ε = 0.00001. The value of ε insured that the limit at *λ*′ = *λ* was evaluated correctly, and *δ* avoided the singularity at *λ*′ = 0 without influencing the value of the integral. The integration was performed with Matlab^@^ code *quadgk*. The solid trace in Fig. 9 shows the result obtained from Eq. (9) using the imaginary index of refraction given in Eq. (7). The expected contribution of the dye to the real part of the microsphere index of refraction was of the order of 0.6 % at the wavelength of maximum absorption.

## 4. Measurement of the Absorbance Due to Microsphere Scattering

**Fig. 10:**
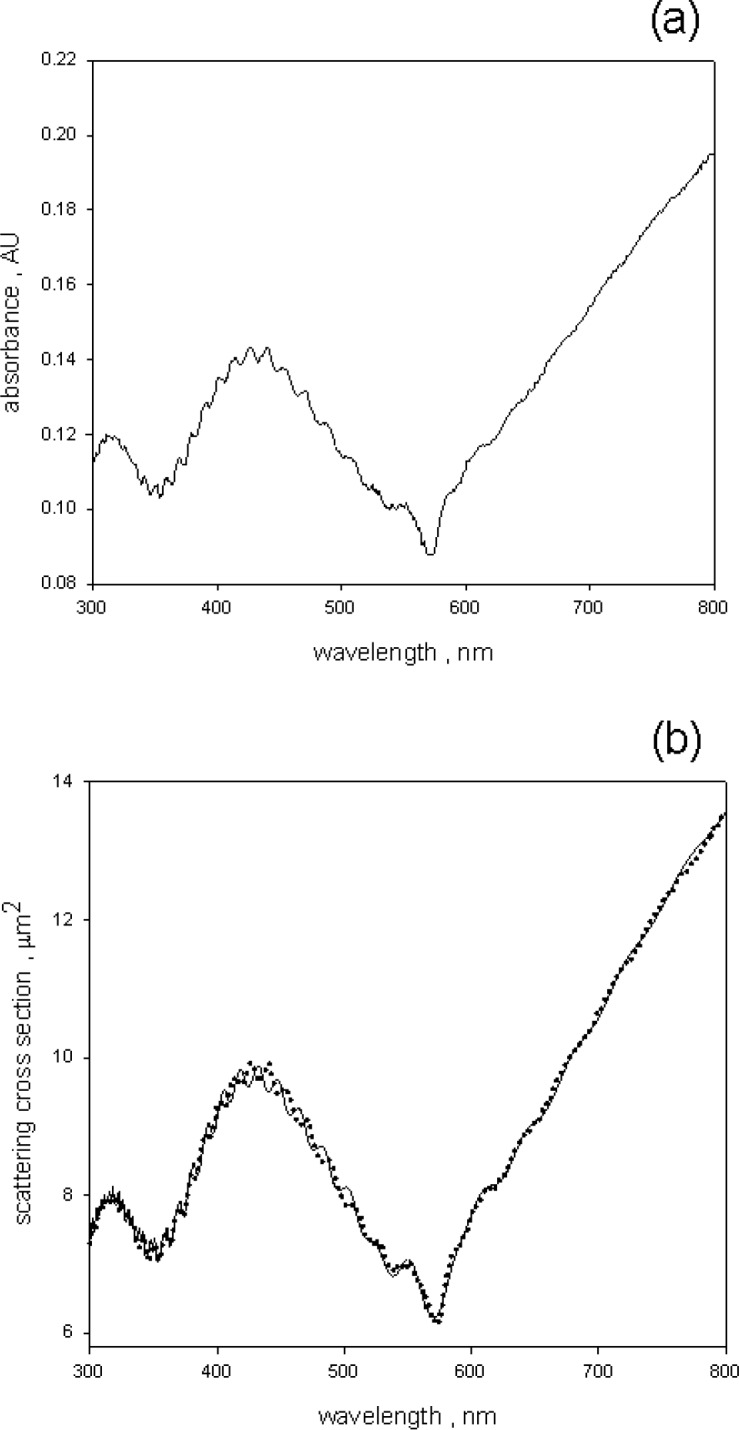
(a) The solid trace (labeled *A_1_-A* in the text) shows the result of subtracting the corrected microsphere absorbance shown in Fig. 8 from the microsphere absorbance measured in holder 1. The effect of BODIPY^@^ dye is apparent in the region around 571 nm. (b) The solid circles are reproduced from (a) and the solid line is the best fit to the data. The calculation used the imaginary index obtained from the analysis of the absorbance (Fig. 8), and varied all of the other parameters to get the best correspondence with the measurements. The parameter c defined in Eq. (10) gave the number concentration of the microspheres.

The fluorescence-corrected absorbance *A*, shown by the solid trace in Fig. 7, was subtracted from the absorbance measured in holder 1, *A_1_*. Figure 10a shows the result of the subtraction. The trace in Fig. 10a, called *A_1_-A* below, represents the absorbance due to scattering. The absorbance due to the dye absorption, shown by the solid trace in Fig. 7, is much smaller than the total absorbance due to scattering and absorption so that when the *A* is subtracted from *A_1_* there was only a minor effect in the absorbance due to scattering at the maximum absorption of the dye. The trace *A_1_-A* in Fig. 10a was analyzed by adding the imaginary index of refraction given by Eq. (7) and the increment in the real index of refraction given by Eq. (9) to the total microsphere index of refraction. The analysis is discussed below.

The solid trace in Fig. 10a was analyzed by minimizing the squares of the deviations from the Lorenz-Mie scattering cross section calculations. The explicit algorithm is shown in Eq. (10). The real part of the index of refraction and the diameter were free to vary, but as mentioned above, the imaginary part of the index of refraction was set to the value given by Eq. (7) with *scale* equal to 0.0078. The best fit to the scattering absorbance, *A_1_ – A*, yielded the microsphere diameter, the real part of the index of refraction of the microsphere, the detector acceptance angle, and the microsphere concentration.

(10) $$\begin{array}{l} Residuals=\sum_{\lambda }\left(\frac{A_{1}-A}{c}-Mie(d,n)+\int_{0}^{\Delta }\frac{d\sigma }{d\theta }(d,n,\theta )d\theta \right)\\ \;c=N\cdot 0.01/2.303\\ \;d=diameter\\ \;n=complex\;index\;of\;refraction\\ \;\Delta =accep\tan ce\;angle \end{array}$$

The detailed explanation of the terms in Eq. (10) were given in reference [1].The best fit is shown by the solid trace in Fig. 10b, the dotted trace in Fig. 10b is reproduced from Fig. 10a. A good fit was obtained only if the contribution of BODIPY given by Eq. (7) was added to the real part of the microsphere index of refraction parameterized as *n_real_* (*λ*) =*A + B* / *λ*^2^ + *C/λ*^4^. To be specific, the best fit gave a scattering cross section of 6.21 µm^2^ compared to the measured value of 6.17 µm^2^ at 571 nm. If the imaginary contribution from BODIPY^@^ was neglected, the calculated scattering cross section was 7.51 µm^2^, which is well outside the estimated uncertainty in the measured cross section (about 6 %) in the region around 571 nm. Adding the BODIPY^@^ contribution to the real part of the index of refraction, *n_real_* (*λ*) defined above, changed the calculated scattering cross section to 6.21 µm^2^. As expected, the inclusion of the real part given by Eq. (9) had a minor effect. The fit was poor in the region around 450 nm where the phases of the ripple structures in the data and the fit do not match. The ripple structure in absorbance has been discussed by Bohren and Huffman [15]. They attributed the ripple structure to the existence of normal modes inside the microsphere. For a homogeneous sphere with a sharp boundary, the ripples and the larger undulations are predicted by the same values of the index of refraction and diameter. In previous measurements [1] the predicted ripple phase and the large undulations matched the data. The fact that in this case the ripples do not match the data suggests that there may be another microsphere property which plays a role in describing the scattering. Perhaps there are changes at the microsphere surface which would affect the normal modes but would not affect the larger undulations which depend mainly on the microsphere diameter.

The best fit to the data, gave a microsphere diameter of 2.47 μm, and a microsphere concentration of 0.63*10^6^ cm^−3^. The value of the real part of the index of refraction and the acceptance angle were consistent with previous observation of PS microspheres. The small decrease in the measured scattering cross section at 571 nm was reproduced by including an imaginary component of the index of refraction obtained from the analysis of trace *A* and the additional real part given by Eq. (9).

## 5. Conclusion

The analysis of the absorbance measured outside (called *A_1_*) and inside (called *A_3_*) of an integrating sphere (IS) detector of a spectrophotometer permitted the identification of absorbance due to microsphere scattering, and the absorbance due to absorption by the BODIPY^@^ dye inside the microsphere. A crucial part of the analysis was the correction of the measured absorbance inside the IS detector for the emission of fluorescence. The correction also yielded an estimate of the microsphere fluorescence quantum yield (QY) which was found to be 0.94±0.05, virtually identical to the value of BODIPY^@^ dye in ethanol. The corrected absorbance, *A*, was subtracted from *A_1_* to give the absorbance due to scattering alone. The analysis of the scattering absorbance, *A_1_-A*, and the absorbance, *A*, was carried out in an iterative manner. It was assumed that the absorbance measured inside the IS, *A*, depended primarily on the imaginary part of the microsphere index of refraction with the other parameters playing a secondary role. Therefore *A* was analyzed first using values of the other parameters obtained from a fit to the absorbance due to scattering, *A_1_-A*, with the imaginary part neglected. The imaginary part obtained from the analysis of *A* was then used to reanalyze *A_1_-A*, and obtain better estimates of the other parameters. After a few iterations, consistent estimates were obtained of the scattering and absorption cross sections in the wavelength region 300 nm to 800 nm. The best fit to *A_1_-A* (shown in Fig. 10b) gave the diameter of the microspheres, the real part of the index of refraction, and the microsphere number concentration. The best fit to *A* (shown in Fig. 8) gave an estimate of the imaginary part of the microsphere index of refraction. The work suggests that it is possible to give a reasonably complete description of the photo physical properties of dyed microspheres which fluoresce in the visible wavelength range.

## Footnotes

1 Certain commercial equipment, instruments, and materials are identified in this paper to foster understanding. Such identification does not imply recommendation or endorsement by the National Institute of Standards and Technology.
